# Emerging Horizons in the Diagnosis of Pancreatic Cancer: The Role of Circulating microRNAs as Early Detection Biomarkers for Pancreatic Ductal Adenocarcinoma

**DOI:** 10.7759/cureus.53023

**Published:** 2024-01-26

**Authors:** Ibrahim Reyaz, Bilal Khan, Neha James, Hammad Azhar, Abdur Rehman, Muhammad Waqas Younas, Hamza Rashid, Faisal F Al-Shaikhly, Mazin M Almomani, Mohammed Khaleel I. KH. Almadhoun, Noor Abdullah Yahya, Syed Faqeer Hussain Bokhari, Ahsan Shehzad

**Affiliations:** 1 Internal Medicine, Christian Medical College and Hospital Ludhiana, Ludhiana, IND; 2 Internal Medicine, Jinnah Postgraduate Medical Centre, Karachi, PAK; 3 General Medicine, Rehman Medical Institute, Peshawar, PAK; 4 Accident and Emergency, Sahiwal Teaching Hospital, Sahiwal, PAK; 5 General Medicine, King Edward Medical University, Lahore, PAK; 6 Surgery, Mayo Hospital, Lahore, PAK; 7 General Medicine, Faisalabad Medical University, Faisalabad, PAK; 8 Medicine, Pak Medical Centre & Hospital, Peshawar, PAK; 9 Medicine and Surgery, University of Jordan, Amman, JOR; 10 Medicine and Surgery, The University of Jordan, Amman, JOR; 11 Medicine and Surgery, Mutah University, Karak, JOR; 12 Department of Medicine, Dubai Medical College, Dubai, ARE; 13 Surgery, King Edward Medical University, Lahore, PAK

**Keywords:** diagnostic accuracy, early detection, biomarkers, mirnas, micrornas, pdac, pancreatic ductal adenocarcinoma

## Abstract

Pancreatic ductal adenocarcinoma (PDAC) is a highly aggressive cancer with a poor prognosis, primarily due to a late diagnosis. Recent studies have focused on identifying non-invasive biomarkers for early detection, with microRNAs (miRNAs) emerging as promising candidates. This systematic review aims to evaluate the potential of circulating miRNAs as biomarkers for the early detection of PDAC, analyzing their diagnostic accuracy, specificity, and sensitivity. Following Preferred Reporting Items for Systematic Reviews and Meta-Analyses (PRISMA) guidelines, a comprehensive search across PubMed, Embase, and the Cochrane Library was conducted. Studies published from January 2013 to October 2023 focusing on miRNA biomarkers for early PDAC detection were included. Data synthesis was performed through a narrative approach due to the heterogeneity of the studies. Nine studies met the inclusion criteria. Key findings include the elevated levels of specific miRNAs, such as miR-18a, miR-106a, and miR-25, in early-stage PDAC patients compared to controls. The integration of miRNA profiles with traditional biomarkers like CA19-9 showed improved diagnostic performance. However, challenges in the standardization of miRNA evaluation methodologies were noted. Circulating miRNAs demonstrate significant potential as non-invasive biomarkers for early PDAC detection. Despite promising results, further research and standardization are necessary for clinical application.

## Introduction and background

Pancreatic ductal adenocarcinoma (PDAC) is a highly aggressive and often fatal form of pancreatic cancer with a notoriously low survival rate. Early detection is crucial for improving patient outcomes, as the majority of cases are diagnosed at an advanced stage when treatment options are limited. In recent years, microRNAs (miRNAs) have emerged as promising candidates for biomarkers in the early detection of PDAC. MiRNAs are small, non-coding RNA molecules that play a key role in the regulation of gene expression. They have been implicated in various cellular processes, including proliferation, differentiation, and apoptosis. The dysregulation of miRNAs has been observed in various cancers, including PDAC, making them potential biomarkers for early detection. Several studies have investigated the clinical application of miRNAs in the early detection of PDAC [1-3]. These studies have explored the expression profiles of miRNAs in PDAC tissues and adjacent normal tissues, identifying specific miRNAs that show differential expression patterns. Notable miRNAs, such as miR-1246, miR-4644, miR-3976, and miR-4306, have been proposed as potential biomarkers for early detection [2].

Furthermore, the diagnostic value of blood-derived miRNAs for pancreatic cancer has been explored, highlighting the potential of miRNAs as non-invasive biomarkers for early detection [4]. Several studies have investigated the diagnostic and prognostic significance of miRNAs in PDAC. A systematic review by Park et al. explored molecular prognostic markers in pancreatic cancer, emphasizing the potential of miRNAs as novel blood-based markers for PDAC diagnosis [5]. Zhao et al. evaluated serum miRNA-192 and identified its diagnostic and biological significance in PDAC, shedding light on its potential as a biomarker [6]. Ma et al. conducted a comprehensive meta-review comparing miRNA expression profiles in PDAC tissues and adjacent normal tissues, providing valuable insights into candidate miRNA biomarkers for PDAC diagnosis [2]. Nesteruk et al. focused on extracellular vesicle-derived miRNAs in pancreatic juice, suggesting their role as biomarkers for the detection of PDAC [7]. In a pilot study by Kane et al., a diagnostic test for PDAC based on the differential expression of select miRNAs in plasma and bile was proposed, highlighting the potential of blood-based biomarkers for PDAC diagnosis [8]. These studies collectively contribute to our understanding of the diagnostic accuracy and potential clinical utility of miRNAs in PDAC.

This systematic review aims to consolidate and analyze the existing evidence on miRNA biomarkers in PDAC, considering their diagnostic accuracy, specificity, and sensitivity. Understanding the role of miRNAs in the early detection of PDAC is not only essential for advancing our knowledge of the disease but also holds great promise for the development of minimally invasive diagnostic strategies. A comprehensive analysis of the available literature will contribute to identifying robust miRNA biomarkers that could revolutionize the early diagnosis and subsequent management of PDAC.

## Review

Materials and methods

This systematic review rigorously follows the established Preferred Reporting Items for Systematic Reviews and Meta-Analyses (PRISMA) guidelines for 2020. The authors employed a comprehensive approach to investigate the potential miRNA biomarkers for early detection of pancreatic ductal adenocarcinoma (PDAC). The subsequent sections delineate the criteria for study inclusion, the search strategy utilized, and the methodology employed for data synthesis.

Search Strategy

A meticulous search strategy was implemented across prominent electronic databases, including PubMed, Embase, and the Cochrane Library, to identify relevant articles. The search comprised a combination of Medical Subject Headings (MeSH) terms and keywords related to pancreatic ductal adenocarcinoma, microRNAs, early detection, and biomarkers. Boolean operators (AND, OR) were strategically employed to refine the search and identify studies meeting the predetermined inclusion criteria. The final search strategy used was "(Biomarkers OR Tumor Markers OR Biomolecular Markers) AND (Early Detection of Cancer OR Early Diagnosis OR "Screening) AND (Pancreatic Neoplasms OR Pancreatic Ductal Carcinoma OR PDAC OR Pancreatic cancer)".

Eligibility Criteria

Stringent inclusion criteria were predefined to ensure the selection of high-quality and relevant studies. The included studies focused on investigating miRNA biomarkers for the early detection of PDAC. Only articles published in peer-reviewed journals within the timeframe of January 2013 until October 2023 were considered. Exclusion criteria encompassed studies on other interventions, those lacking sufficient data on miRNA biomarkers, and studies solely involving animal cells. Additionally, only studies in the English language with full-text availability were included, and gray literature was not considered eligible.

Data Extraction and Synthesis

Two independent reviewers meticulously screened titles and abstracts to identify potentially eligible studies. Subsequently, full-text articles were retrieved and evaluated for adherence to inclusion criteria. Discrepancies between reviewers were resolved through discussion and consultation with a third reviewer. Relevant data, including study design, patient characteristics, miRNA biomarkers investigated, and outcomes, were systematically extracted using a predefined data extraction form.

Data Analysis

A narrative synthesis approach was employed to summarize findings from included studies due to anticipated heterogeneity in study designs and outcome measures. Key themes and patterns related to miRNA biomarkers for the early detection of PDAC were identified and presented.

This rigorous methodology ensures a systematic and transparent evaluation of the existing literature, laying a robust foundation for synthesizing evidence regarding miRNA biomarkers in the early detection of PDAC.

Results

Study Selection Process

**Figure 1:**
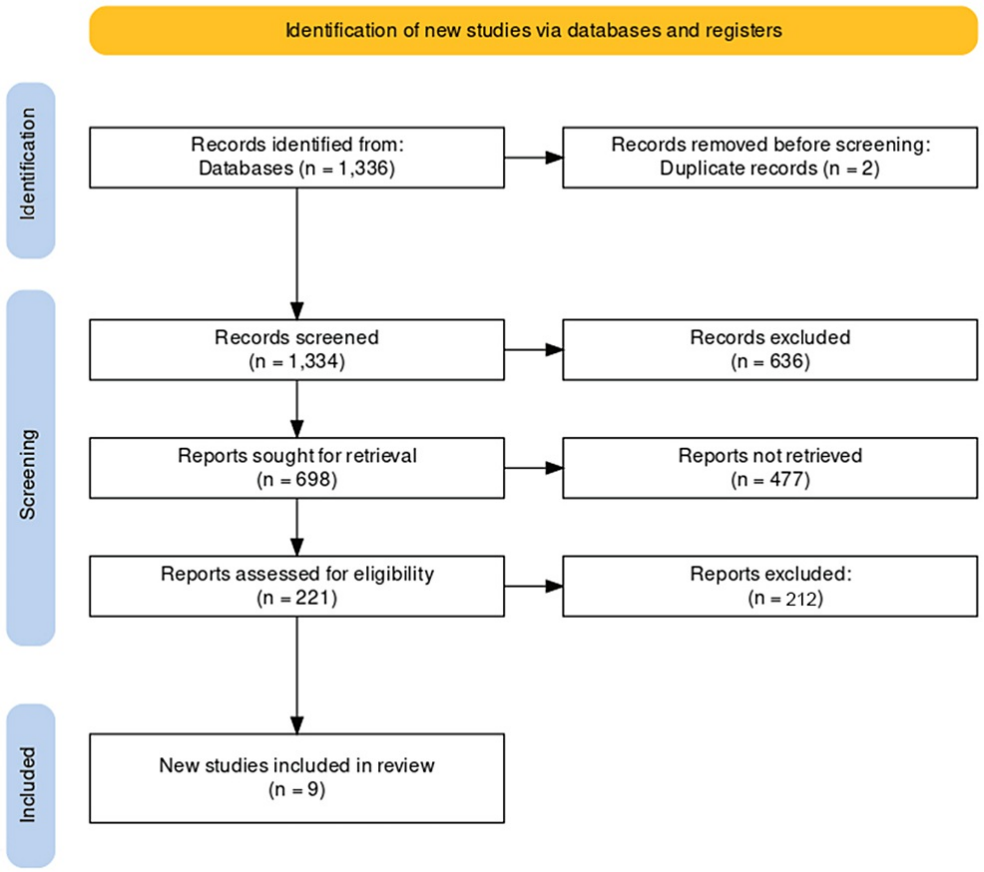
PRISMA flow diagram of selection of the studies for inclusion in the systematic review PRISMA: Preferred Reporting Items for Systematic Reviews and Meta-Analyses

Following database searches, 636 articles were initially identified. After eliminating 2 duplicates, the titles and abstracts of the remaining 634 publications were evaluated. Subsequently, 30 potential studies underwent eligibility verification through a thorough examination of their full texts. Ultimately, nine articles satisfied the inclusion criteria. No additional studies meeting the eligibility criteria were found during the examination of references in the selected articles. The entire process is visually depicted in the PRISMA flowchart (Figure 1).

Characteristics of Selected Studies

Overall, nine papers met the inclusion criteria. Six studies were cross-sectional studies, two were cohorts, and one was an in vitro cell culture study. Three studies each were from France, China, and the United States. The main findings and characteristics of the included studies are mentioned in Table 1. The quality of the included studies was assessed using the Newcastle-Ottawa quality assessment scale (Table 2).

**Table 1:** A summary of the studies included in this systematic review miR: MicroRNA; EVs: Small Extracellular Vesicles; PDAC: Pancreatic Ductal Adenocarcinoma; IPMN: Intraductal Papillary Mucinous Neoplasm; AUC: Area Under the Receiver Operating Characteristic Curve; LCN2: Lipocalin 2; TIMP1: Tissue Inhibitor of Metalloproteinases 1; IAR: Individuals at Risk; CEA: Carcinoembryonic Antigen; ROC: Receiver Operating Characteristic; mRNA: Messenger RNA; lncRNAs: Long Non-Coding RNAs; ceRNA: Competing Endogenous RNA; DLCN: Dysregulated lncRNA-associated ceRNA Network; LncRisk-7: 7-lncRNA Signature; SVM: Support Vector Machine; RTqPCR: Real-Time Quantitative Polymerase Chain Reaction; USA: United States of America

Author	Year	Country	Study Type	Biomarkers studied	Main Findings
Xu et al. [9]	2023	USA	Cross-sectional	miR-18a and miR-106a in plasma small EVs	The levels of miR-18a and miR-106a were significantly elevated in plasma EVs of early-stage PDAC patients compared to controls. This indicates these miRNAs are promising biomarkers for early PDAC detection.
Vila-Navarro et al. [10]	2019	Spain	Cross-sectional	A set of 17 miRNAs was previously identified as overexpressed in pancreatic neoplasms. These include miR-21-5p, miR-33a-3p, miR-320a, and miR-93-5p, among others. Additionally, the study also examined CA19-9 levels.	A set of 17 circulating miRNAs significantly overexpressed in PDAC and IPMN patients compared to controls. Combinations of miR-33a-3p, miR-320a, and CA19-9 levels had high diagnostic accuracy in distinguishing pancreatic neoplasms from controls.
Dittmar et. al. [11]	2023	USA	Cross-sectional	Circulating miRNAs, specifically miR-34a-5p, miR-130a-3p, and miR-222–3p, as well as CA19-9.	miR-34a-5p, miR-130a-3p, and miR-222-3p were identified as significant miRNA biomarkers for detecting early-stage PDAC. Combining these miRNAs with CA19-9 improved diagnostic performance.
Bartsch et. al. [12]	2018	Germany	Cross-sectional	miRNA-196b, LCN2 (Lipocalin 2), TIMP1 (Tissue Inhibitor of Metalloproteinases 1), Glypican-1, RNU2-1f, and KRAS mutations.	miR-196b, LCN2, TIMP1, and KRAS mutations accurately distinguished precursor lesions and early-stage PDAC. These biomarkers were elevated in high-risk individuals with pancreatic lesions.
Deng et al. [13]	2016	China	Cross-sectional	miR-25	Serum miR-25 levels were significantly higher in PDAC patients versus controls. MiR-25 outperformed CEA and CA19-9 as a diagnostic marker.
Gong et al. [14]	2023	China	Cohort	miR-25, CA19-9, CEA, CA125	miR-25 and CA19-9 had the highest diagnostic accuracy for PDAC detection compared to CEA and CA125. Their combination further improved sensitivity.
Sakai et al. [15]	2019	Japan	Cross-sectional	mRNA expression levels of 56 genes in whole blood	The study developed a blood mRNA PDAC screening system with a sensitivity of 73.6% and specificity of 64.7% for noncancer subjects. It was effective for early-stage PDAC diagnosis.
Zhou et al. [16]	2016	China	Cohort	lncRNAs, miRNAs, and mRNAs	The study constructed a dysregulated lncRNA-associated ceRNA network and developed a 7-lncRNA signature for early PDAC diagnosis, validated in multiple cohorts.
Makler et al. [17]	2022	USA	In vitro Cell Culture	Exosomal miRNAs: miR-31-5p, miR-31-3p, miR-210-3p, miR-339-5p, miR-425-5p, miR-425-3p, and miR-429	7 exosomal miRNAs were identified as potential early detection biomarkers for PDAC.

**Table 2:** Quality assessment of the included studies using the Newcastle-Ottawa quality assessment scale

Study	Selection	Comparability	Outcome
Xu et al. [9]	★★★★	★★	★★★
Vila-Navarro et al. [10]	★★★★	★★	★★★
Dittmar et. al. [11]	★★★★	★★	★★★
Bartsch et. al. [12]	★★★★	★★	★★★
Deng et al. [13]	★★★	★	★★★
Gong et al. [14]	★★★	★★	★★★
Sakai et al. [15]	★★★★	★★	★★★
Zhou et al. [16]	★★★	★	★★★
Makler et al. [17]	★★★	★	★★★

Discussion

The landscape of pancreatic cancer diagnosis is undergoing a transformative shift with the emerging focus on circulating miRNAs as potential biomarkers. Our study, in alignment with recent research, sheds light on the rising significance of these circulating miRNAs in the early detection of PDAC. We observed that miR-18a and miR-106a in plasma small extracellular vesicles (EVs) were significantly elevated in early-stage PDAC patients compared to healthy controls. This finding aligns with other studies, suggesting these miRNAs as reliable indicators for early PDAC detection [3,9,18-20]. The consistency across different research efforts reinforces the potential of these miRNAs as non-invasive biomarkers.

The differential expression of miRNAs between PDAC, intraductal papillary mucinous neoplasms (IPMNs), and healthy individuals provides a compelling avenue for non-invasive diagnosis [4,21-23]. Our findings, along with others, indicate a similar miRNA expression pattern between tissue and plasma, suggesting that these circulating cancer-associated miRNAs might originate directly from tumor cells or as a response to carcinogenesis [21-23]. The identification of specific miRNAs that are upregulated in PDAC but not in premalignant IPMNs is particularly intriguing, as it hints at their potential role in the malignant progression of IPMNs [24,25].

The integration of circulating miRNAs with traditional biomarkers like CA19-9 has been shown to enhance the diagnostic accuracy of PDAC [26-28]. Our study supports this approach, demonstrating that the addition of CA19-9 to miRNA signatures improves overall diagnostic performance. This synergy suggests that a multi-biomarker approach could be more effective for early detection and screening of pancreatic neoplasia [23]. However, an important consideration is that CA19-9 levels can be influenced by non-cancer factors such as pancreatitis and biliary obstruction [29]. Therefore, combining miRNA profiles with CA19-9 could help offset the limitations of relying solely on CA19-9.

A critical challenge highlighted in our study and others is the need for standardization in miRNA evaluation methodologies [17,30-32]. The current landscape is marked by variability and low reproducibility across different studies and laboratories. Standardizing protocols and techniques is imperative for advancing these biomarkers from research to clinical application. Achieving this would require consensus guidelines established through multi-disciplinary expert working groups. Pipeline optimization considering sample processing, miRNA extraction, profiling platform, and data normalization is also vital [33,34].

Our focus on biomarkers for detecting PanINs, IPMNs with advanced dysplasia, and early-stage PDAC in the context of FPC is particularly relevant. The biomarker set comprising miR-196b, LCN2, and TIMP1 showed promise in distinguishing individuals with significant precursor lesions and early-stage PDAC from healthy controls [12]. This finding is crucial for screening in high-risk populations such as those with a family history of pancreatic cancer. However, further validation studies are required before translating this set into clinical practice for FPC surveillance.

While our findings are promising, they are not without limitations. The small sample sizes and the need for validation in larger cohorts are significant constraints. Future research should focus on elucidating the biological roles of these miRNAs in PDAC and developing robust plasma miRNA biomarker panels [35-38]. Additionally, exploring the functional role of these miRNAs in circulation is critical for their advancement as suitable biomarkers [39]. Consideration of demographic factors and health history that could influence miRNA profiles would also strengthen the analysis [40,41].

In summary, our study contributes to the growing body of evidence supporting circulating miRNAs as valuable biomarkers for early detection of PDAC. The potential for these non-invasive biomarkers to improve early diagnosis and prognosis of this lethal disease is significant. However, further research, standardization, and validation are necessary to fully realize their clinical potential.

## Conclusions

This systematic review underscores the potential of circulating microRNAs (miRNAs) as non-invasive biomarkers for the early detection of pancreatic ductal adenocarcinoma (PDAC). Our analysis, alongside current research, highlights specific miRNAs, such as miR-18a, miR-106a, and miR-25, which show differential expression in PDAC patients compared to healthy individuals. These findings suggest their significant utility in early cancer diagnosis. A notable advancement is the integration of miRNA profiles with traditional biomarkers like CA19-9, enhancing diagnostic accuracy. This multi-biomarker approach could be especially beneficial for high-risk groups, such as those with familial pancreatic cancer, where early detection is critical. However, our review also identifies a crucial need for standardization in miRNA evaluation methodologies. The current variability in research protocols underscores the importance of establishing uniform methods to ensure consistent and reliable results across studies. Despite promising developments, limitations such as small sample sizes and the necessity for broader validation remain. Future research should aim to deepen our understanding of the biological roles of these miRNAs in PDAC and their functional implications in circulation, which is essential for their transition from research to clinical application.
